# Haplotypic structure of the X chromosome in the COGA population sample and the quality of its reconstruction by extant software packages

**DOI:** 10.1186/1471-2156-6-S1-S77

**Published:** 2005-12-30

**Authors:** Fabio Marroni, Chiara Toni, Benedetto Pennato, Ya-Yu Tsai, Pryia Duggal, Joan E Bailey-Wilson, Silvano Presciuttini

**Affiliations:** 1 Center of Statistical Genetics, c/o Centro Retrovirus, SS Abetone e Brennero 2, 56127 Pisa, Italy; 2 Unit of Legal Medicine, University of Pisa, Pisa, Italy; 3 Center for Inherited Disease Research, NHGRI, NIH, Baltimore, USA

## Abstract

**Background:**

The haplotypes of the X chromosome are accessible to direct count in males, whereas the diplotypes of the females may be inferred knowing the haplotype of their sons or fathers. Here, we investigated: 1) the possible large-scale haplotypic structure of the X chromosome in a Caucasian population sample, given the single-nucleotide polymorphism (SNP) maps and genotypes provided by Illumina and Affimetrix for Genetic Analysis Workshop 14, and, 2) the performances of widely used programs in reconstructing haplotypes from population genotypic data, given their known distribution in a sample of unrelated individuals.

**Results:**

All possible unrelated mother-son pairs of Caucasian ancestry (*N *= 104) were selected from the 143 families of the Collaborative Study on the Genetics of Alcoholism pedigree files, and the diplotypes of the mothers were inferred from the X chromosomes of their sons. The marker set included 313 SNPs at an average density of 0.47 Mb. Linkage disequilibrium between pairs of markers was computed by the parameter D', whereas for measuring multilocus disequilibrium, we developed here an index called D*, and applied it to all possible sliding windows of 5 markers each. Results showed a complex pattern of haplotypic structure, with regions of low linkage disequilibrium separated by regions of high values of D*. The following programs were evaluated for their accuracy in inferring population haplotype frequencies: 1) ARLEQUIN 2.001; 2) PHASE 2.1.1; 3) SNPHAP 1.1; 4) HAPLOBLOCK 1.2; 5) HAPLOTYPER 1.0. Performances were evaluated by Pearson correlation (*r*) coefficient between the true and the inferred distribution of haplotype frequencies.

**Conclusion:**

The SNP haplotypic structure of the X chromosome is complex, with regions of high haplotype conservation interspersed among regions of higher haplotype diversity. All the tested programs were accurate (*r *= 1) in reconstructing the distribution of haplotype frequencies in case of high D* values. However, only the program PHASE realized a high correlation coefficient (*r *> 0.7) in conditions of low linkage disequilibrium.

## Background

With the advent of dense genetic maps of single-nucleotide polymorphisms (SNPs), large population samples of diallelic multilocus genotypes are increasingly available for studies in the fields of population genetics, marker-disease association, and evolutionary genetics. However, current genotyping methods do not provide information on the individual diplotypes (the haplotype pair composing a genotype). This information would nonetheless increase the power of any genetic analysis substantially. Several methods for estimating haplotype frequencies from a sample of genotyped but unphased diploid individuals have been developed. These include a sequential haplotype inference algorithm [1], several expectation-maximization based algorithms [2-4], a coalescent-based algorithm based on a Markov chain Monte Carlo approach [5], a Bayesian approach that uses a Dirichlet prior distribution for the haplotype frequencies [6], and a recent method based on Bayesian networks that takes account of recombination hotspots, bottlenecks, genetic drift, and mutation [7]. The X chromosome is unique as a population genetics tool because of its diploidy in females and haploidy in males, a characteristic that, among other things, renders its haplotypes accessible to direct count. The potential of the X chromosome to contribute to fine-scale microevolutionary studies (which are dominated by mtDNA and the Y chromosome) has probably been underused [8].

The purposes of the present work were dual. First, we wanted to ascertain whether a haplotype structure could be detected on the X chromosome in a Caucasian population sample, given the genotypes provided by Illumina and Affimetrix for Genetic Analysis Workshop 14. Second, we wanted to test and compare the capability of widely used programs in reconstructing haplotypes, given their distributions in a sample of individuals with known diplotypes; for this purpose, the haplotypes in a sample of unrelated mothers (treated as independent individuals) were determined using the data on their sons, so that the accuracy of different methods of inferring haplotypes from genotype data could be evaluated by comparing the true and the inferred distributions of haplotype frequencies.

## Methods

All possible unrelated mother-son pairs of Caucasian ancestry were selected from the 143 families of the Collaborative Study on the Genetics of Alcoholism pedigree files. The diplotypes of the mothers were inferred from the X chromosomes of their sons. An integrated map of the X chromosome for the Illumina and Affymetrix SNP datasets was obtained by querying the NCBI Human Genome (Build 34) for marker position. The Affimetrix dataset was first cleared of the markers with heterozygosity <0.2 in order to render the 2 datasets more homogeneous. The final map included 313 markers (121 Illumina, 192 Affimetrix). It spanned 146.5 Mb at an average density of 0.47 Mb. A large gap (7.8 Mb) was located at 56.5 Mb. Linkage disequilibrium (LD) between pairs of markers was computed by the parameter D'. For multilocus disequilibrium, we defined here an index called D*. This is computed as D* = 1 - (H_d _- H_min_)/(H_eq _- H_min_), where the haplotype diversity H_d _is computed as H_d _= 1 - ∑ p_i_^2^, (p_i _being the frequency of haplotype *i*, analogous to the gene diversity of a single locus), the expected haplotype diversity under no LD, H_eq_, is calculated as H_eq _= 1 - ∑ E{p_i_}^2 ^(E{p_i_} being the expected frequency of each possible haplotype, i.e., the product of the frequencies of the alleles composing that haplotype), and the minimum possible value of haplotype diversity H_min _is obtained computationally. Specifically, if *n *haplotypes typed for *s *SNPs are arranged in a *n *by *s *matrix and the alleles are coded consistently (e.g., 0 = low frequency allele at all loci), H_min _is obtained by computing H_d _with the above equation, after the matrix is rearranged by sorting iteratively each column independently of the others. Another measure of multilocus disequilibrium (the normalized entropy difference, ε) was published previously [9]. We applied both D* and ε to all possible sliding windows of 5 SNPs each.

The following programs were evaluated for their accuracy in inferring population haplotype frequencies: 1) ARLEQUIN 2.001; 2) PHASE 2.1.1; 3) SNPHAP 1.1; 4) HAPLOBLOCK 1.2; 5) HAPLOTYPER 1.0. In inferring haplotypes, ARLEQUIN and SNPHAP use an expectation-maximization algorithm, HAPLOTYPER uses a Bayesian approach assuming a prior Dirichlet distribution of haplotype frequencies, PHASE uses a coalescence-based algorithm for inferring the prior distribution of haplotype frequencies coupled with a Markov chain Monte Carlo approach to approximate the posterior distribution, and HAPLOBLOCK uses a Bayesian network method. ARLEQUIN and SNPHAP ignore the missing data, whereas PHASE and HAPLOTYPER make informed guesses; in HAPLOBLOCK, users can choose between these 2 options. Among all programs, only PHASE include the possibility of specifying a genetic map and modeling the process of recombination. Genotypes at 5 or 10 consecutive markers were selected from the Illumina dataset based on varying levels of D*, and were submitted to all programs. The accuracy of each program was measured using the Pearson correlation coefficient between the true and the inferred haplotype frequencies.

## Results

**Figure 1:**
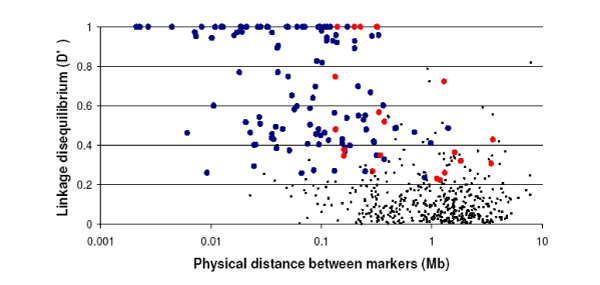
**Standardized linkage disequilibrium as a function of intermarker distance**. Standardized linkage disequilibrium (D') between markers of the X chromosome as a function of the intermarker distance. Large symbols: D' values with nominal *p *≤ 0.01 (blue: adjacent markers; red: LD computed at 5-marker intervals). Dots: D' values with p > 0.01. Marker pairs with distance <1 kb have been omitted.

**Figure 2:**
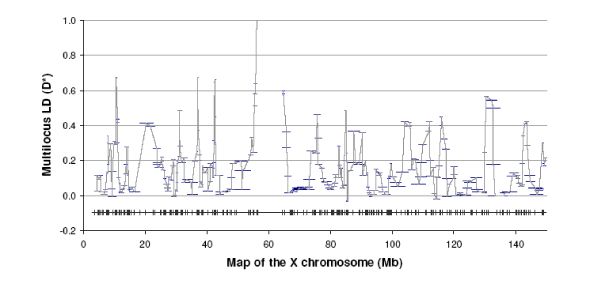
**Multilocus LD of the X chromosome**. Bars represent sliding windows of 5 markers each, whose D* value is plotted. The line under the chart shows the marker location; a large gap centered at 60 Mb may be noted.

The final dataset analyzed in the present work consisted of 104 unrelated Caucasian females with known diplotypes at 313 SNPs on the X chromosome. Figure 1 shows the parameter D' between all adjacent markers and between each marker and the fifth marker downstream; 84% of marker pairs closer than 100 kb showed high levels of LD, with *p*-values < 0.01, in comparison with 34% of the pairs 100 to 500 kb apart, and 4.4% of the pairs 500 kb to 2 Mb apart. Two marker pairs with an intermarker distance >3 Mb showed highly significant LD. Then, the multilocus haplotype structure of the X chromosome was investigated by considering sliding windows of 5 markers and calculating both the parameters D* and ε. The 2 measures were highly correlated (*r *= 0.945). Because of the uneven marker distribution in the maps, the length of the 5-marker segments was highly variable, from 93 kb to 7.94 Mb; in the present analysis, segments longer than 5 Mb were not considered. Several regions of high values of D* (low haplotype diversity) were separated by segments with similar values of H_d _and H_eq _(no LD, Figure 2). One instance of D* = 1.0 (i.e., in which H_d _= H_min_) was located at about 56 Mb, near the large gap in the chromosome map. These 5 markers were part of a chromosome segment of 10 markers spanning 1.33 Mb for which only 7 haplotypes, out of 1,024 theoretically possible, were observed. The value of D* for this segment of 10 markers was 0.74. This is consistent with previous reports of a substantial recombination decrease in the centromere of the X chromosome [10].

Fourteen series of unphased genotypes with different values of D* (10 series consisting of 5 consecutive markers and 4 of 10 markers) were submitted to each of the 5 programs. Table 1 shows Pearson correlation coefficients between the observed and the inferred haplotype frequencies. For 5-marker haplotypes, the correlation coefficients were high even in situations of moderate LD for all programs. In the case of 10-marker haplotypes (last 4 rows in Table 1), all the programs reconstructed perfectly well the true haplotype distribution when the number of different haplotypes in the sample was small in comparison with the total number of possible haplotypes. With the increase of haplotype diversity (series 11 in Table 1), the performance of the programs started to decrease and differentiate, though the correlation between the true and the estimated haplotype frequencies was still high; PHASE realized the best performance (*r *= 0.996). In the opposite situation, when the haplotype diversity was high (130 different haplotypes in a sample of 104 individuals) the performances were generally poor; only PHASE realized a high correlation coefficient (0.737). When the majority of the haplotypes is unique (last row in Table 1), the inferred haplotypes are clearly unreliable.

## Discussion

We investigated the large-scale haplotypic structure of the X chromosome in a Caucasian population sample by computing D' for all adjacent markers and any fifth marker; high levels of LD were detected even at distances > 1 Mb. We then applied to all possible segments of 5 consecutive markers a measure of multilocus LD, here called D*. This parameter is easily computed and is based on the standard definition of heterozygosity; D* reaches its maximum possible value of 1.0 when the haplotype diversity is at a minimum, i.e., when LD is complete. Thus, D* appeared to be a suitable measure in studies of large-scale multilocus linkage disequilibrium. In addition, we wanted to test the capability of widely used programs in reconstructing the haplotypes of population samples. All investigated programs perform well when the number of markers is small (5) even in situations of low values of D*. With a higher number of markers (10), high correlation values between true and inferred haplotype frequencies are attained only in conditions of high D*. PHASE is an exception, in that it has reconstructed the true distribution of haplotype frequency with good accuracy even in a difficult situation. This program employed significantly more computing time than the others (10–20 minutes in comparison with less than a second using in the same machine), with the exception of HAPLOBLOCK, which ran for more than 30 hours.

## Conclusion

The SNP haplotypic structure of the X chromosome is complex, with regions of high haplotype conservation (most notably, around the centromere) interspersed among regions of higher haplotype diversity. A more detailed definition of this structure, to be accomplished in further studies, could be useful in evolutionary analyses and in disease association studies.

All the tested programs were accurate (*r *= 1) in reconstructing the true distribution of haplotype frequencies in case of high LD. Only the program PHASE realized a high correlation coefficient (*r *> 0.7) in case of low linkage disequilibrium.

## Abbreviations

LD: Linkage disequilibrium

SNP: Single-nucleotide polymorphism

## Authors' contributions

FM participated in all phases of statistical analyses and drafted the manuscript. CT participated in the analysis of 2-locus and multilocus linkage disequilibrium. BP participated in the analyses of software performances. Y-T integrated the SNP maps. PD selected the families to be used in the study. JEB-W participated in the study conception, and provided critical revision of the manuscript for important intellectual content. SP conceived of the study, participated in its design and coordination, and helped to draft the manuscript. All authors read and approved the final manuscript.

## Figures and Tables

**Table 1:** Correlation between the true haplotype frequencies and those estimated by five programs

	Series	Location^a^	Length^b^	#Hap^c^	D*	Arlequin (EM)^d^	Phase (CP)^e^	Snp Hap (EM)	Haplo Block (BN)^f^	Haplo typer (D)^g^
5-marker haplotypes	1	55,992,117	0.14	3	1	1	1	1	1	1
	2	10,464,050	0.14	10	0.6	1	1	1	1	1
	3	54,499,735	1.42	5	0.59	1	1	1	1	1
	4	71,732,319	2.62	8	0.46	1	1	1	1	1
	5	36,765,524	0.13	11	0.36	1	1	1	1	1
	6	84,938,832	1.46	9	0.28	0.97	0.99	0.97	0.98	0.89
	7	42,260,770	4.25	20	0.19	0.95	0.99	0.96	0.95	0.92
	8	90,806,576	2.23	16	0.18	0.94	0.96	0.94	0.83	0.92
	9	63,668,482	3.84	30	0.05	0.84	0.95	0.82	0.75	0.77
	10	3,562,850	3.86	31	0.02	0.75	0.91	0.75	0.67	0.65

10-marker haplotypes	11	54,499,735	1.63	6	0.74	1	1	1	1	1
	12	55,920,880	10.51	15	0.44	0.99	1	0.98	0.93	0.97
	13	74,309,896	10.79	130	0.02	0.51	0.78	0.50	0.07	0.50
	14	14,437,620	13.36	165	0.02	0.14	0.35	0.19	0.25	0.17

^a^nucleotide position of the first marker of the haplotype

^b^length of the haplotype (Mb)

^c^number of different haplotypes in the sample

^d^EM: expectation-maximization algorithm

^e^CP: coalescent prior

^f^BN: Bayesian networks

^g^D: Dirichlet prior

## References

[B1] Clark AG (1990). Inference of haplotypes from PCR-amplified samples of diploid populations. Mol Biol Evol.

[B2] Excoffier L, Slatkin M (1995). Maximum-likelihood estimation of molecular haplotype frequencies in a diploid population. Mol Biol Evol.

[B3] Hawley ME, Kidd KK (1995). HAPLO: a program using the EM algorithm to estimate the frequencies of multi-site haplotypes. J Hered.

[B4] Long JC, Williams RC, Urbanek M (1995). An E-M algorithm and testing strategy for multiple-locus haplotypes. Am J Hum Genet.

[B5] Stephens M, Smith NJ, Donnelly P (2001). A new statistical method for haplotype reconstruction from population data. Am J Hum Genet.

[B6] Niu T, Qin ZS, Xu X, Liu JS (2002). Bayesian haplotype inference for multiple linked single-nucleotide polymorphisms. Am J Hum Genet.

[B7] Greenspan G, Geiger D (2004). High density linkage disequilibrium mapping using models of haplotype block variation. Bioinformatics.

[B8] Schaffner SF (2004). The X chromosome in population genetics. Nat Rev Genet.

[B9] Nothnagel M, Furst R, Rohde K (2002). Entropy as a measure for linkage disequilibrium over multilocus haplotype blocks. Hum Hered.

[B10] Mahtani MM, Willard HF (1998). Physical and genetic mapping of the human X chromosome centromere: repression of recombination. Genome Res.

